# Attending to what’s important: what heat maps may reveal about attention, inhibitory control, and fraction arithmetic performance

**DOI:** 10.3389/fpsyg.2023.1210266

**Published:** 2023-11-01

**Authors:** Karrie E. Godwin, Clarissa A. Thompson, Freya Kaur, Yuika Iwai, Charles J. Fitzsimmons, Jennifer M. Taber

**Affiliations:** ^1^Department of Psychology, University of Maryland, Baltimore County, Baltimore, MD, United States; ^2^Sherman Center for Early Learning in Urban Communities, University of Maryland, Baltimore County, Baltimore, MD, United States; ^3^Department of Psychological Sciences, Kent State University, Kent, OH, United States; ^4^Department of Psychology, University of North Florida, Jacksonville, FL, United States

**Keywords:** attention, inhibitory control, executive functions, fractions, math

## Abstract

Math proficiency is an important predictor of educational attainment and life success. However, developing mathematical competency is challenging, and some content (e.g., fractions) can be enigmatic. Numerous factors are suspected to influence math performance, including strategy knowledge, attention, and executive functions. In two online studies, we investigated the relationship between adults’ fraction arithmetic performance, confidence judgments, inhibitory control (a component of executive functions), and attention to strategy-relevant fraction components. We explored the utility of heat maps (based on mouse clicks) to measure adults’ attention to strategy-relevant fraction arithmetic components (operationalized according to each mathematical operation). In Study 1, attending to strategy-relevant fraction components was correlated with inhibitory control, but this finding did not replicate in Study 2. Across both studies, inhibitory control and attention to strategy-relevant fraction components were correlated with arithmetic accuracy. Intraindividual variability in participants’ attention to strategy-relevant fraction components was also found. Our findings suggest that heat map questions may be a viable alternative to assess participants’ attention during fraction tasks and that attention to specific fraction-arithmetic problem features is related to problem-solving accuracy.

## Introduction

Being adept in mathematics is advantageous in education and daily life (e.g., health, finances, cooking). However, math is difficult, and math competency involves a complex interplay of subject knowledge and general cognitive processes, including executive functions (EF). In a series of two studies, we used heat maps, a question format in the Qualtrics survey platform, to examine whether participants’ self-reported attention to strategy-relevant components of fraction arithmetic equations (i.e., numerators, denominators, operations) was associated with participants’ EF and fraction arithmetic performance.

Greater competency in mathematics is associated with higher educational attainment, greater financial security, and more life satisfaction (National Mathematics Advisory Panel, 2008; Bjälkebring and Peters, 2021). Unfortunately, many U.S. adults and children lack mathematical competency [U.S. Department of Education, Institute of Education Sciences, National Center for Education Statistics, National Assessment of Educational Progress (NAEP), 2022]. One source of difficulty in math competency is developing an understanding of fractions (Booth and Newton, 2012; Siegler et al., 2013; Siegler, 2016a). Difficulties with fractions may be compounded by math anxiety and negative math attitudes about fractions relative to other types of numbers (Mielicki et al., 2021; Sidney et al., 2021) which may hinder performance (Mielicki et al., in press).

Prior research suggests people tend to report greater confidence in their performance when they are accurate, reflecting some degree of metacognitive accuracy (Siegler and Pyke, 2013). However, an inverse relationship between confidence and performance is also possible; for example, if an individual has a misconception, they may have high confidence in their response and yet exhibit low accuracy (Bercher, 2012; Dunlosky and Rawson, 2012; Nelson and Fyfe, 2019). Furthermore, people’s confidence in their performance on fraction tasks may not be aligned with their accuracy on these tasks (Fitzsimmons et al., 2020a; Rivers et al., 2021; Scheibe et al., 2022). For instance, even when women and men were equally accurate in their placement of fractions on number lines, men were more confident than women about their performance on this task (Rivers et al., 2021). Incongruity between math performance and confidence may have important downstream effects. For example, low math confidence may be related to female college students’ decisions to leave the “STEM Pipeline” (Ellis et al., 2016). Given the importance of mathematical competency and confidence for numerous indicators of life success, it is important to elucidate obstacles to developing competency in mathematics as well as to identify potential cognitive factors (e.g., attention, executive functions) that can support learning and be leveraged in future interventions to help remediate deficits in fraction understanding.

Given the inherent difficulty of developing competency in math and an understanding of fractions specifically, it is perhaps unsurprising that *both* children and adults make frequent errors when asked to solve fraction arithmetic problems (Siegler et al., 2011; Siegler and Pyke, 2013; Braithwaite and Siegler, 2018a; Di Lonardo Burr et al., 2020). Examining people’s self-reported strategies for solving math problems (i.e., “strategy reports”) can provide insight into the types of errors participants make. According to the Dynamic Strategy Choice Account (Alibali and Sidney, 2015; Fitzsimmons et al., 2020b; see also Siegler, 2016b), errors participants make when solving fraction arithmetic problems vary as a function of the operation type and the problem features, such as whether the fractions have a common denominator. For example, one error individuals make when solving addition and subtraction problems with unlike denominators is to neglect to compute the common denominator and instead mistakenly operate directly *across* numerators and denominators (e.g., incorrectly claiming that ¾ + ⅖ = 5/9). Another type of error individuals make is to apply a problem-solving strategy to the wrong operation. For instance, individuals may err in their understanding of when to use the strategy of inverting the second operand, a strategy used when dividing fractions.

### Cognitive skills and math performance

As discussed above, gaps in foundational mathematical knowledge, math anxiety, and math confidence may all contribute to the difficulties individuals experience developing mathematical competencies. In addition, math performance may also be influenced by domain general cognitive skills including attention and executive functions, which we discuss briefly below.

#### Attention and math performance

Attention is a limited cognitive resource in which only a small subset of information can be selected for further processing (see Chun et al., 2011; Oberauer, 2019). Attention regulation can occur exogenously, driven by the environment or features of a stimulus (e.g., novelty), or endogenously in a goal-directed manner (see Pashler et al., 2001; Ruff and Rothbart, 2001; Corbetta and Shulman, 2002). The ability to control attention endogenously is thought to be especially important for learning (Erickson et al., 2015).

Attention has been linked with performance on a variety of mathematical competencies and measures (e.g., Benedetto-Nasho and Tannock, 1999; Steele et al., 2012; Anobile et al., 2013; LeFevre et al., 2013; Antonini et al., 2016; Capodieci and Martinussen, 2017). For example, Li et al. (2023) found that attention regulation mediated the relationship between children’s math anxiety and performance. Children with higher math anxiety had difficulty inhibiting distractions and were found to attend more to a visual distractor presented during the math task. Greater attention to the distractor was in turn predictive of lower performance on the math task.

The ability to selectively attend to and maintain a focused state of attention over time is also likely important for successful strategy selection. For example, in a fraction arithmetic task, individuals who have difficulty regulating their attention to relevant fraction components may be less likely to identify and deploy a strategy that is aligned with the given problem (e.g., not attending to the denominators when subtracting fractions with uncommon denominators). However, to our knowledge, it remains an open question whether individual differences in attention regulation are related to strategy selection and performance and in particular with performance on fraction arithmetic problems. Work integrating eye tracking as well as performance based measures of attention will help elucidate these questions.

#### Executive function and math performance

Executive function (EF) refers to the cognitive processes that guide or control goal-directed behavior (Best and Miller, 2010). EF is considered a multidimensional construct and is often conceptualized as three separate but related cognitive functions, which include inhibitory control, updating/monitoring working memory, and cognitive flexibility/shifting (Miyake et al., 2000). Although EF emerges early in development, improvements with age are noted during childhood (Zelazo and Müller, 2002). EF is thought to continue to develop into adolescence (Best and Miller, 2010), with declines in EF noted in late adulthood (Zelazo et al., 2004).

EF is related to academic achievement and may be particularly important for performance in mathematics and reading (St Clair-Thompson and Gathercole, 2006; McClelland et al., 2007; Kieffer et al., 2013; Cragg and Gilmore, 2014; Cortés Pascual et al., 2019, for review see Zelazo and Carlson, 2020). For example, a meta-analysis (Jacob and Parkinson, 2015) found that EF was correlated with math (average correlation = 0.30) and reading (average correlation = 0.31) achievement in children and teens (3–18 years).

There is reason to believe that EFs likely support people’s math performance in multiple ways. When solving problems, people may rely on their working memory to hold intermediate steps in mind. For example, when comparing, adding, or subtracting fractions, individuals may need to first find a common denominator. One approach to finding a common denominator is to generate a list of multiples for each denominator, holding the multiples in memory until a common multiple is determined. When solving various math problems, people may rely on their cognitive flexibility/shifting to switch among several known strategies. For example, when solving intermixed fraction addition and division problems, individuals need to flexibly switch between strategies in order to deploy a strategy that aligns with the given operation (Fazio et al., 2016; Sidney et al., 2019b).

The role of inhibitory control on math performance has recently garnered interest (Siegler and Pyke, 2013; Gilmore et al., 2015). Indeed, the ability to inhibit prepotent (i.e., automatically deployed, readily available in memory) responses may be particularly important when reasoning about fractions (e.g., Gomez et al., 2015). Individuals may struggle to focus on the magnitude of a fraction as opposed to attending in isolation to integers as they are accustomed to when working with whole numbers (Ni and Zhou, 2005; Siegler et al., 2011; Alibali and Sidney, 2015). For instance, when working with the fraction 5/6, individuals may focus erroneously on the individual components of the fraction (e.g., 5 and 6) as opposed to the magnitude of the fraction (e.g., 0.833). Similarly, individuals may generalize whole-number operations to fractions and add numerators or denominators to produce an answer rather than adding the magnitudes of the fraction addends (Siegler and Lortie-Forgues, 2015). As we noted in the example above, individuals may operate directly *across* numerators and denominators; and thus incorrectly conclude that 3/4 + 2/5 = 5/9. If the magnitude of the fractions are not taken into account, participants may fail to realize that their answer of 5/9 (or 0.555) is necessarily incorrect as its magnitude is *less* than just one of the addends (3/4 = 0.75). Thus, an individual’s ability to inhibit this prepotent integer-based strategy when solving fraction arithmetic problems may be important for accurate performance (e.g., Siegler and Pyke, 2013). Further, performance on a numerical Stroop task, a measure of inhibitory control, has been associated with participants’ ability to effectively reason about fractions. Fitzsimmons et al. (2020b) presented participants with a series of numerical stimuli. For each test item, participants were instructed to compare the physical size of the numerals while ignoring their magnitude. Individuals with better inhibitory control, indexed by their performance on the Stroop task, were also more accurate on fraction estimation and magnitude comparison problems (*rs* ≥ 0.55). Other work found that executive function skills (indexed by an antisaccade task and a working memory task) are positively related to fraction arithmetic performance (Siegler and Pyke, 2013). We build on this work and assess whether inhibitory control is related to fraction arithmetic performance by employing a domain-specific measure, the numerical Stroop task (Fitzsimmons et al., 2020b).

### Approaches to measurement

Measuring participants’ understanding of fractions, and conversely their misconceptions, has been done in a variety of ways: assessing participants’ accuracy (Thompson and Opfer, 2008; Fazio et al., 2014; Siegler and Thompson, 2014), response times (Fazio et al., 2014), open-ended strategy reports (Siegler et al., 2011; Fazio et al., 2016; Sidney et al., 2019b; Fitzsimmons et al., 2020b; Thompson et al., 2021a,b), and fMRI (Wortha et al., 2020), among other approaches. Each of these approaches to measurement can provide important insights about an individual’s conceptual understanding. In seminal work investigating fraction number-line estimation, magnitude comparison, and fraction arithmetic (Siegler et al., 2011), open-ended strategy reports, in which participants were asked to describe how they solved each problem, were correlated strongly with accuracy on fraction tasks. However, as with all measurement approaches, open-ended strategy reports are not without their limitations. Open-ended strategy reports may require that an individual possess the metacognitive abilities to accurately reflect on, and then verbally articulate, their strategies. In addition, coding open-ended strategy reports is time consuming, and it can be difficult to establish strong inter-rater reliability.

Eye tracking technology has also been used to measure participants’ attention to fraction components and infer their strategy use during comparison (Hurst and Cordes, 2016; Obersteiner and Tumpek, 2016). Eye movements and gaze duration are sometimes used to infer areas of a display that capture participants’ attention and the mental operations participants are completing (e.g., Schneider et al., 2008; Obersteiner and Tumpek, 2016). Additionally, the ongoing COVID-19 pandemic has necessitated modifications to researchers’ data collection approaches, yielding considerable interest and urgency in creating tools that can also be leveraged to collect data remotely (Nunes, 2020; Waldroff, 2020; Fyshe and Werker, 2021; Kubota, 2021). Eye-tracking data can be difficult to collect in online settings where there are often greater demands on participants to upload their data, equity issues given the equipment required to participate (e.g., access to webcam, reliable internet connection), and additional burden on researchers to oversample due to concerns with data loss (Bott et al., 2017; Semmelmann and Weigelt, 2018). In pivoting to online data collection, one fruitful approach is to assess whether existing technologies can be adapted to successfully capture participants’ attention and infer their mathematical strategies. This study provides an initial exploration of the utility of the heat map function in Qualtrics to index participants’ attention and problem-solving strategies in a fast and low-cost way. In addition, this work aims to provide support for the Dynamic Strategy Choice Account by capturing which fraction components participants report attending to (see Method section for additional details).

### Current study

In this work, we explored five questions: (1) does fraction arithmetic accuracy vary as a function of operation type, (2) is fraction arithmetic accuracy related to adults’ confidence judgments, (3) is inhibitory control related to adults’ fraction arithmetic accuracy, (4) is self-reported attention, indexed via heat maps, to different fraction components (i.e., numerators, denominators, operations) related to inhibitory control, fraction arithmetic accuracy, and confidence judgments, and (5) do these findings replicate?

Research questions 1 and 2 were largely confirmatory in nature and intended to establish that participants’ performance is aligned with the prior literature. Based on prior research, we anticipated that fraction arithmetic accuracy would vary as a function of operation type (Siegler et al., 2011; Braithwaite et al., 2017, 2019; Sidney and Alibali, 2017; Braithwaite and Siegler, 2018b; Sidney et al., 2019a) and that fraction arithmetic accuracy would be associated with participants’ confidence judgments (Siegler and Pyke, 2013). Regarding research question 3, we hypothesized that adults with better inhibitory control, indexed by a numerical-Stroop task, would be more accurate in a fraction-arithmetic task as both measures are correlated with performance on estimation and magnitude comparison tasks (Siegler et al., 2011). Our examination of the utility of heat maps to measure attention to fraction components and strategy use was largely exploratory (Research question 4). However, we anticipated that attention to the operation and to relevant fraction arithmetic components, operationalized by the specific mathematical operation (i.e., addition/subtraction, multiplication, division) and indexed by participants’ mouse clicks, would be positively correlated with participants’ inhibitory control and fraction arithmetic performance. Study 2 provided a first step in assessing the replicability of the results from Study 1 (Note that the larger parent study from which the data for Study 1 was obtained is a pre-registered study: https://osf.io/ywu8b?view_only=9b0bab92ad424f05862eae26d6b05379. See page 9 of the Study 1 PDF preregistration for information on the collection of heat map data from the arithmetic task. Data for Study 2 was also part of a larger pre-registered parent study: https://osf.io/ztukp?view_only=c02f248837dc406a862dcde2184ba549). To facilitate comparisons across studies, we present the results from Study 1 and 2 together.

## Method

### Participants

In Study 1, adult participants were recruited via an online platform, Prolific. Forty-one participants were excluded from analysis due to at least one of the following exclusion criteria: completing less than 75% of the study, failing both attention checks, providing open-ended responses that were gibberish, and/or completing the study in less than ⅓ (i.e., 20 min) of the estimated completion time or more than 3*SD* longer than the mean time of completion. The final sample for Study 1 included 379 participants. Of the participants who reported their demographic information, a little more than half (55.9%) self-identified as female, and the majority of participants self-identified as White (73.9%). The majority of participants reported having an associates degree or higher (66%). Most participants (70.7%) were employed, and 58.7% had incomes of $74,999 or less.

Study 2 also included adult participants who were recruited via Prolific. The same exclusion criteria used in Study 1 were employed in Study 2. Based on these criteria, 49 participants were excluded from the analysis. The final sample for Study 2 included 306 participants. Of the participants who reported their demographic information, a little more than half of the participants self-identified as males (56.6%), and the majority of participants identified as White (72.8%). The majority of participants reported having an associates degree or higher (66.7%). Of those who reported their employment status, the majority of participants were employed (76.6%), and 65.5% had incomes of $74,999 or less. See Table 1 for full details on the participant demographics from each study.

**Table 1:** Participant demographic information for Study 1 and Study 2.

	Study 1			Study 2		
	*N*	%	Valid%	*N*	%	Valid%
**Gender**						
Male	157	41.4	41.8	171	55.9	56.6
Female	210	55.4	55.9	123	40.2	40.7
Non-binary/third gender	9	2.4	2.4	7	2.3	2.3
Other	0	0	0	1	0.3	0.3
Did not report	3	0.8	–	4	1.3	–
**Education level**						
Less than high school graduate	2	0.5	0.5	5	1.6	1.6
High school graduate/GED	51	13.5	13.6	47	15.4	15.4
Some college or trade school	75	19.8	19.9	50	16.3	16.3
Associate degree	23	6.1	6.1	38	12.4	12.4
Bachelor’s degree	161	42.5	42.8	118	38.6	38.6
Graduate degree	64	16.9	17.0	48	15.7	15.7
Did not report	3	0.8	–	0	0	–
**Child race/ethnicity**						
American Indian/Alaska native	0	0	0	0	0	0
Asian	27	7.1	7.2	26	8.5	8.6
Black/African American	26	6.9	6.9	25	8.2	8.3
Hispanic or Latino	20	5.3	5.3	12	3.9	4.0
Native Hawaiian/Pacific Islander	0	0	0	0	0	0
White	278	73.4	73.9	220	71.9	72.8
Two or more	25	6.6	6.6	18	5.9	6.0
Other	0	0	0	1	0.3	0.3
Did not report	3	0.8	–	4	1.3	–
**Employment**						
Employed for wages	214	56.5	58.0	182	59.5	60.9
Self-employed	47	12.4	12.7	47	15.4	15.7
Student	47	12.4	12.7	12	3.9	4.0
Out of work for more than a year	24	6.3	6.5	28	9.2	9.4
Out of work for less than a year	15	4.0	4.1	10	3.3	3.3
Homemaker	15	4.0	4.1	6	2.0	2.0
Retired	7	1.8	1.9	14	4.6	4.7
Did not report	10	2.6	–	7	2.3	–
**Income level**						
<15,000	20	5.3	5.5	29	9.5	9.9
15,000–24,999	37	9.8	10.2	26	8.5	8.9
25,000–34,999	29	7.7	8.0	36	11.8	12.3
35,000–49,999	59	15.6	16.3	46	15.0	15.7
50,000–74,999	68	17.9	18.7	55	18.0	18.8
75,000–99,999	67	17.7	18.5	46	15.0	15.7
100,000–149,000	55	14.5	15.2	34	11.1	11.6
150,000–199,999	16	4.2	4.4	12	3.9	4.1
>/=200,000	12	3.2	3.3	9	2.9	3.1
Did not report	16	4.2	–	13	4.2	–

### Procedure

Participants completed a series of online tasks via Qualtrics assessing their fraction arithmetic performance, item-level confidence, self-reported attention, and inhibitory control. Participants completed additional tasks as part of a larger parent study examining the relationship between math performance and health decisions. Note that within Qualtrics responses were not forced. Thus, participants could elect to skip individual items and thus the sample size per item varies. The study was largely self-paced and on average participants spent 68.29 min (SD = 24.49 min) completing Study 1 and 63.65 min (SD = 21.79 min) completing Study 2.

This research was approved by the Kent State University Institutional Review Board (IRB; # 17-432). Participants read the online consent form and indicated electronically whether they agreed to participate. Participants were compensated with $13 for their participation in the study.

### Measures

#### Fraction arithmetic performance

Participants completed 24 fraction arithmetic problems (6 problems per operation type: addition, subtraction, multiplication, division; Siegler and Pyke, 2013). Eight of these problems (2 per operation type) served as critical trials and assessed participants’ attention to various fraction components and their fraction strategies. Half of the critical trials had a common denominator (e.g., 3/5 + 4/5), whereas the other half had a different denominator (e.g., 2/3 + 3/5). A full list of arithmetic problems is included in the see Supplementary material. Participants were asked to solve each fraction arithmetic problem and then to type their answer into response boxes (i.e., numerator and denominator response boxes). The presentation order of items was blocked by operation type. Non-responses were scored as incorrect. Additionally, for this study, participants were not asked to reduce their answer to the lowest term and therefore, answers that were not reduced were still deemed to be correct, as were correct answers that were written as mixed fractions or decimals. For a more detailed description of the scoring protocol, please see the Supplementary material. We calculated the mean accuracy for: all 24 fraction arithmetic problems, the eight critical trials, and separately by operation type.

#### Self-report attention measure: selection of operation and strategy-specific AOIs via mouse click

**Figure 1:**
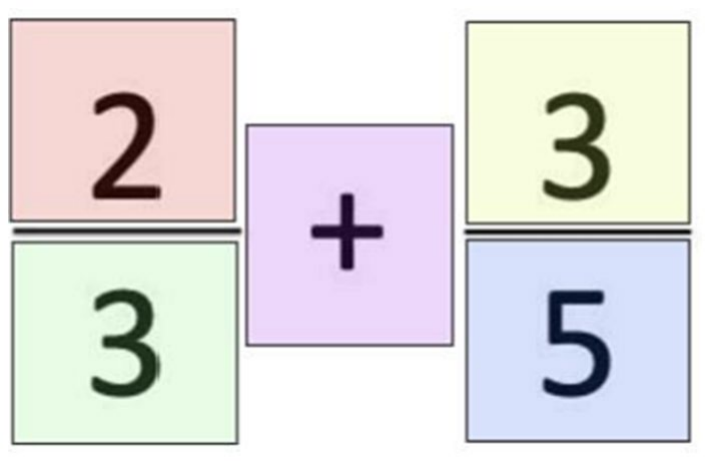
Schematic of key Areas of Interest (AOIs) (Numerator 1 [top left], Numerator 2 [top right], Denominator 1 [bottom left], Denominator 2 [bottom right], Operation [middle]) used for the heat map analysis of the self-report attention measure. The AOIs were not visible to participants.

As mentioned previously, there were eight critical trials (adapted from Siegler and Pyke, 2013) that were designed specifically for the current studies to assess participants’ attention to specific fraction components and their fraction strategies by incorporating the Qualtrics heat map function. For each critical trial, participants were asked to select the part of the problem they attended to first, via mouse click. The Qualtrics’ heat map function was used to code whether participants reported attending to one of six Areas of Interest (AOIs): Numerator 1 (top left), Numerator 2 (top right), Denominator 1 (bottom left), Denominator 2 (bottom right), Operation (middle), and Other (anywhere else on screen); see Figure 1. The AOIs were not visible to participants so as not to bias their attention to a particular part of the equation. For this study, participants were only able to make a single selection, via mouse click, of where they attended to first. Therefore, we were only able to assess the first part of the problem participants attended to, but not the sequential problem-solving order. Note that in Study 1 due to a technical error for one of the critical trials, 37 participants were able to make multiple mouse clicks, and therefore, their responses for this critical trial were excluded from the analysis. Additionally, if participants did not provide a response for a particular item(s), their mean selection AOI scores were calculated based only on participants’ available responses. On average, 84% (SD = 28%) of the critical trials were completed in Study 1 and 89% (SD = 23%) in Study 2.

We anticipated that participants would attend to the operation first to help them determine which strategy to use to solve the fraction arithmetic problems. Thus, we calculated the mean number of times participants reported attending first to the Operation AOI (i.e., Operation AOI score) across the eight critical trials.

We also created an average Operation-specific strategy score (i.e., Strategy-Specific AOI score) for the critical trials, in which we coded whether participants reported attending to specific components of the fraction arithmetic problem that aligned with an operation-specific strategy. The Strategy-Specific AOI score was hypothesized to demonstrate the next steps in a multi-step strategy after identifying the operation type, which could be used to solve fraction arithmetic problems. For all operation types, selection of any non-strategy specific AOIs were coded as 0 (i.e., having not selected a Strategy-Specific AOI) and selection of Strategy-Specific AOIs were coded as 1. The Strategy-Specific AOIs were defined for each operation as follows: For addition/subtraction problems, if participants selected either denominator AOI (i.e., Denominator 1 or 2), it was scored as 1 (i.e., having selected a Strategy-Specific AOI), as participants need to assess whether they must calculate a common denominator (Siegler et al., 2011). For multiplication problems, participants who reported attending to either numerator AOI (i.e., Numerator 1 or 2) were scored as 1, based on the common strategy of multiplying first across numerators (Siegler et al., 2011). For division problems, participants who selected either Numerator 2 or Denominator 2 were scored as 1, based on the common strategy of invert and multiply (Sidney et al., 2022). Due to the limited number of critical trials per operation type (2 problems each), we decided to focus our analysis of the self-report attention measure on participants’ average Strategy-Specific AOI scores across all eight critical trials, rather than by operation type.

#### Confidence judgments

After completing each problem, participants were asked to rate their confidence in their answer on a trial-by-trial basis (e.g., Wall et al., 2016; Fitzsimmons et al., 2020a; Rivers et al., 2021; Fitzsimmons and Thompson, 2022, 2023; Scheibe et al., 2022) on a scale from 0 to 100, with higher scores indicating greater confidence. We calculated participants’ average confidence score across all fraction arithmetic problems and by operation type.

#### Numerical Stroop task

To assess inhibitory control, participants completed the Numerical Stroop task (Dadon and Henik, 2017; Fitzsimmons et al., 2020b). Participants saw a series of single-digit dyads and were asked to select the physically larger number as quickly and accurately as possible, while ignoring its magnitude. Participants completed three types of trials: incongruent, congruent, and neutral trials. For incongruent trials, the physically larger number was the number that was *smaller* in magnitude (e.g., 1 vs. 2). Whereas, for congruent trials, the physically larger number was also larger in magnitude (e.g., 1 vs. 2). For neutral trials, the magnitude of the two numbers were identical but the physical size of the stimuli differed (e.g., 1 vs. 1). Of the 112 trials that participants completed, 32 trials were incongruent, 32 trials were congruent, and 48 trials were neutral. Due to a technical error in the presentation of the stimuli, two participants were excluded from the analysis.

The analyses focused on reaction time (RT) for incongruent trials (for correct trials only). Greater inhibitory control is indicated by shorter RTs. Prior studies have noted that a RT of 200 ms or less may not be a valid RT, as it would not reflect the entire process of encoding a stimulus and executing a response (Whelan, 2008; Berger and Kiefer, 2021). Therefore, for our analysis, we excluded any trials in which participants’ RTs were 200 ms or less. Additionally, the trials were designed to automatically advance after 2 s; however, due to a technical error, some responses were recorded after 2 s. These responses (i.e., RTs greater than 2 s) were excluded from the analysis as well. As a result of the RT trimming, in Study 1, 274 of 41,725 correct trials (0.66%) were excluded, and in Study 2, 204 trials of 33,372 correct trials (0.61%) were excluded.

To verify that Numerical Stroop RTs varied as a function of trial type (congruent, incongruent, neutral), we conducted 3-way repeated measures ANOVAs (with Huynh-Feldt corrections). As expected, across both Study 1 and 2 there was a significant effect of trial type on participants’ RTs (*Fs* ≥ 787.15; *ps* < 0.001, partial η^2^ ≥ 0.68). Pairwise comparisons confirmed that participants’ RTs (correct trials only) were significantly slower for incongruent trials than for congruent or neutral trials; *ps* < 0.001; indicating that the task was functioning as intended.

### Data analysis plan

To assess whether participants’ fraction arithmetic accuracy varied as a function of operation type, we conducted a 4-way repeated measure ANOVA with follow-up pairwise comparisons. Additionally, we conducted correlation analyses to investigate the association between participants’ fraction arithmetic accuracy (across and within operation types) and their confidence judgments. We also conducted a correlation analysis to explore the relationship between participants’ AOI selections and their fraction arithmetic accuracy and confidence judgments. To assess whether participants’ RT on the Numeric Stroop task (a measure of inhibitory control) varied as a function of item type (congruent, incongruent, neutral) we conducted a 3-way repeated measures ANOVA with follow-up pairwise comparisons. Additionally, we used correlational analyses to assess the association between participants’ inhibitory control (incongruent block RT) and their fraction arithmetic accuracy and AOI selections.

## Results

Descriptive Statistics for both Study 1 and Study 2 are provided in Table 2. Despite participants’ relatively high levels of education, average fraction arithmetic accuracy was fairly low in both studies. Additionally, at the aggregate level, confidence judgments were similar to average fraction-arithmetic accuracy.

**Table 2:** Descriptive statistics [M(SD)] and sample size for Study 1 and Study 2.

	Study 1		Study 2	
	Mean (SD)	*N*	Mean (SD)	*N*
**Fraction arithmetic accuracy**				
All trials	66% (28%)	379	70% (28%)	306
Critical trials	69% (29%)	379	73% (28%)	306
**Confidence judgments**				
All trials	62.78 (28.02)	379	64.39 (29.74)	306
Critical trials	64.57 (29.12)	379	65.85 (30.09)	306
**AOI**				
Operation AOIs	28% (36%)	353	23% (33%)	292
Strategy relevant AOIs	39% (28%)	353	43% (27%)	292
Conservative strategy AOIs	32% (24%)	353	34% (25%)	292
**Stroop accuracy**				
Congruent	97% (9%)	377	98% (7%)	306
Incongruent	93% (12%)	377	95% (9%)	306
Neutral	97% (8%)	377	98% (7%)	306
**Stroop RT**				
Congruent	0.70 s (0.23 s)	377	0.70s (0.22)	305
Incongruent	0.85 s (0.24 s)	376	0.86 s (0.24)	305
Neutral	0.73 s (0.23 s)	377	0.74 s (0.23)	305

The AOI variable refers to the mean percentage of critical trials in which participants reported attending to the Operation AOIs, Strategy relevant AOIs (AOIs aligned to the operation type; e.g., denominator AOIs for addition/subtraction problems) and Conservative strategy AOIs; e.g., denominator 1 [Bottom Left] AOIs for addition/subtraction problems.

### Self-report attention measure

Across the critical trials, selection of the Operation AOI was less common than we anticipated (28% in Study 1 and 23% in Study 2). Selection of the remaining AOI regions was as follows: Numerator 1 (top left) = 20% in Study 1 and 20% in Study 2; Denominator 1 (Bottom left) = 32% in Study 1 and 36% in Study 2; Numerator 2 (top right) = 5% in Study 1 and 4% in Study 2; Denominator 2 (Bottom right) = 11% in Study 1 and 13% in Study 2.

As part of an exploratory analysis, we evaluated the *consistency* with which individuals selected the Operation AOIs and the Strategy-Specific AOIs across the eight critical trials. A consistent Operation responder was defined as an individual who selected the Operation AOI on six or more trials (out of 8 or 75%). Similarly, a Strategy-Specific responder was defined as an individual who selected the Strategy-Specific AOI on six or more trials (out of 8 or 75%). In Study 1, 17% of participants were consistent Operation responders and only 11% were consistent Strategy-Specific responders. In Study 2, 13% of participants were classified as consistent Operation responders and 14% of participants were classified as consistent Strategy- Specific responders. These findings highlight the considerable variability in participants’ self-reported attention to relevant fraction arithmetic components and underscores the importance of increasing the number of test items per operation, a point we return to in the discussion. By increasing the number of trials per operation, we could examine the stability and dynamics of participants’ problem-solving approaches and ascertain whether certain operations are more taxing on inhibitory control (e.g., addition and subtraction with non-common denominators).

### Accuracy on the fraction arithmetic problems

In Study 1, the average accuracy on the 24 fraction arithmetic problems was 66% (SD = 28%). To ascertain whether participants’ performance varied as a function of operation type (i.e., addition, subtraction, multiplication, division) we conducted a 4-way repeated measures ANOVA, with a Greenhouse-Geisser correction, on accuracy scores with operation type as the within-subject factor. Based on prior research with children and adults, we hypothesized that accuracy rates would be the lowest for fraction division problems (Siegler et al., 2011; Braithwaite et al., 2017, 2019; Sidney and Alibali, 2017; Braithwaite and Siegler, 2018b; Sidney et al., 2019a). A significant effect of operation type on mean accuracy scores was found [*F*(2.05, 775.71) = 71.82, *p* < 0.001, partial η^2^ = 0.16]. The results aligned with our hypothesis; pairwise comparisons indicated that accuracy was lower on division problems (*M* = 52%, SD = 42%) compared to all other operation types: addition (*M* = 72%, SD = 30%), subtraction (*M* = 74%, SD = 28%), and multiplication (*M* = 64%, SD = 37%); all *ps* < 0.001. Participants also exhibited higher accuracy scores on addition and subtraction problems compared to multiplication problems (both *ps* < 0.001), and they were also more accurate on subtraction problems in comparison to addition problems (*p* = 0.002).

**Figure 2:**
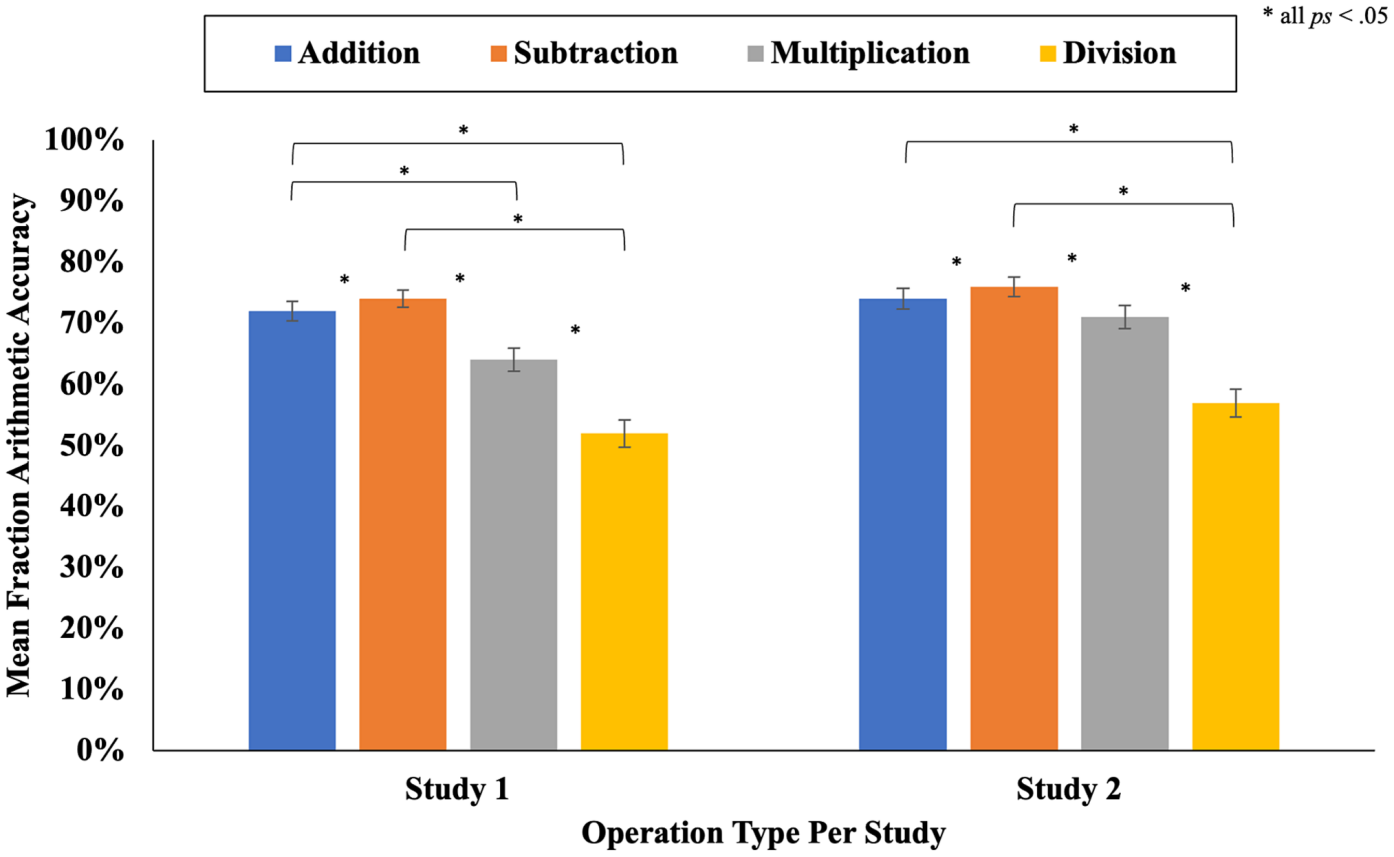
Mean accuracy on fraction arithmetic problems by operation type and study. Error bars represent the standard errors of the means.

In Study 2, participants showed a similar level of accuracy on the fraction arithmetic problems as they did in Study 1 (*M* = 70%, SD = 28%). Also in line with the findings from Study 1, a 4-way repeated measures ANOVA with Greenhouse–Geisser correction revealed a significant effect of operation type on fraction arithmetic accuracy [*F*(2.21, 673.85) = 48.52, *p < 0*.001, partial η^2^ = 0.14]. Pairwise comparisons indicated that participants in Study 2 also exhibited lower accuracy rates on division problems (*M* = 57%, SD = 41%) compared to all other operation types: addition (*M* = 74%, SD = 29%), subtraction (*M* = 76%, SD = 29%), and multiplication (*M* = 71%, SD = 34%); all *ps* < 0.001. Participants also exhibited higher accuracy scores on subtraction problems compared to addition problems and multiplication problems (both *p*s ≤ 0.019). However, unlike Study 1, there was no significant difference in participants’ accuracy scores on addition and multiplication problems (*p* = 0.154); see Figure 2.

### Association between fraction arithmetic accuracy and confidence judgments

Recall that we asked participants to rate their confidence in their fraction arithmetic answers on a scale from 0 to 100 with higher ratings indicating greater confidence. In Study 1, participants’ mean confidence judgment was 62.78 (SD = 28.02). On average, participants who reported greater confidence in their fraction arithmetic answers (across operation types) also tended to exhibit higher accuracy on fraction arithmetic problems (*r* = 0.73, *p* < 0.001). The findings were analogous within operation type (all *rs* ≥ 0.57, all *ps* < 0.001).

Consistent with results in Study 1, in Study 2 fraction arithmetic accuracy was positively correlated with participants’ confidence judgments (*M* = 64.39, SD = 29.74; *r* = 0.68, *p* < 0.001). This pattern of result was unchanged when looking at the association between accuracy and confidence judgments within each operation type (all *rs* ≥ 0.53, all *ps* < 0.001), in which individuals who were more accurate also tended to report greater confidence in their fraction arithmetic answers.

### Association between self-report attention measure and fraction arithmetic accuracy

Here, we focus our correlational analyses on the eight critical fraction arithmetic trials for which participants also completed the self-report attention measure. Recall that for the self-report attention measure, participants reported (via mouse click) the part of the fraction arithmetic problem they attended to first (i.e., operation, numerator, denominator AOIs). In addition, follow-up analyses were also conducted using all fraction arithmetic trials (*N* = 24) to begin testing the consistency of the findings.

We predicted that regardless of problem type, participants would report attending to the Operation AOI given that the operation should cue participants as to which strategy is required to successfully solve each type of fraction arithmetic problem. The variable Operation AOI was determined for each participant by calculating the mean selection of the Operation AOIs across the eight critical trials. In Study 1, the correlation analysis indicated that there was no significant correlation between fraction arithmetic accuracy on critical trials (*M* = 69%, SD = 29%) and participants’ tendency to select the Operation AOI (*M* = 28%, SD = 36%; *r* = 0.01, *p* = 0.84). Even when including all fraction arithmetic trials, this pattern of results was unchanged (*r* = 0.04, *p* = 0.50). The results were analogous in Study 2. The association between participants’ selection of the Operation AOI (*M* = 23%, SD = 33%) and their fraction arithmetic accuracy was not significant when examining both the association with participants’ accuracy on the eight critical trials (*M* = 73%, SD = 28%; *r* = 0.08, *p* = 0.16) and when substituting accuracy scores for all fraction arithmetic items (*r* = 0.08, *p* = 0.18).

This finding was unexpected and counter to our hypothesis. It is possible this finding may be due in part to the design choice to block the presentation of the fraction arithmetic problems by operation type. It is conceivable that blocking by operation type cued participants to attend to other aspects of the problem, besides the operation. This *post-hoc* explanation can be explored in future research.

Next, we conducted correlation analyses to explore the hypothesis that the tendency to select AOIs that are *aligned* to the operation type (i.e., Strategy-Specific AOIs) would be positively correlated with participants’ fraction arithmetic accuracy. Recall that the Strategy-Specific AOI score was calculated across critical trials by calculating for each participant their mean selection of AOIs that were aligned to each problem type (i.e., selecting Denominator 1 or Denominator 2 AOIs for addition/subtraction problems, Numerator 1 or Numerator 2 AOIs for multiplication problems, and Numerator 2 or Denominator 2 AOIs for division problems). In Study 1, the mean selection of Strategy-Specific AOIs (*M* = 39%, SD = 28%) was positively correlated with participants’ mean fraction arithmetic accuracy scores on critical trials (*r* = 0.17, *p* = 0.001). This pattern of results held even after substituting participants’ accuracy for all fraction arithmetic trials (vs. critical trials only); *r* = 0.18, *p* < 0.001. As in Study 1, in Study 2 participants who reported attending to strategy-relevant fraction arithmetic components (*M* = 43%, SD = 27%) also tended to be more accurate on the eight critical fraction arithmetic problems (*r* = 0.24, *p* < 0.001). This finding held when substituting fraction arithmetic accuracy for all 24 fraction problems; *r* = 0.26, *p* < 0.001.

### Association between the self-report attention measure and confidence judgments

To assess whether participants who reported attending *first* to fraction arithmetic components that aligned with a problem-solving strategy relevant to the operation type were also more confident in their fraction arithmetic answers, we conducted a correlation analysis on participants’ mean Strategy-Specific AOI score and their mean confidence judgment score on the eight critical trials. In Study 1, we found a positive correlation between participants’ selection of Strategy-Specific AOIs and their confidence judgments (*M* = 64.57, SD = 29.12; *r* = 0.12, *p* = 0.03) suggesting that participants who reported directing their attention to aspects of the fraction arithmetic problem that were relevant to a strategy that could be used to successfully solve the problem were also more likely to report greater confidence in their answers. This pattern was unchanged after substituting participants’ confidence judgments for all fraction arithmetic trials (vs. critical trials only); *r* = 0.11, *p* = 0.04.

In Study 2, the correlation between participants’ attention to strategy-relevant fraction components and their confidence in their fraction arithmetic answers on the eight critical trials (*M* = 65.85, SD = 30.09) was in the expected direction (i.e., positive); however, it was not statistically significant (*r* = 0.10, *p* = 0.09). The results were unchanged when substituting participants’ confidence judgment scores for all fraction arithmetic trials, *M* = 64.39, SD *=* 29.74*, r = 0*.09, *p =* 0.11.

### Inhibitory control: numerical Stroop task

#### Association between inhibitory control (Stroop RT) and self-report attention measure

**Figure 3:**
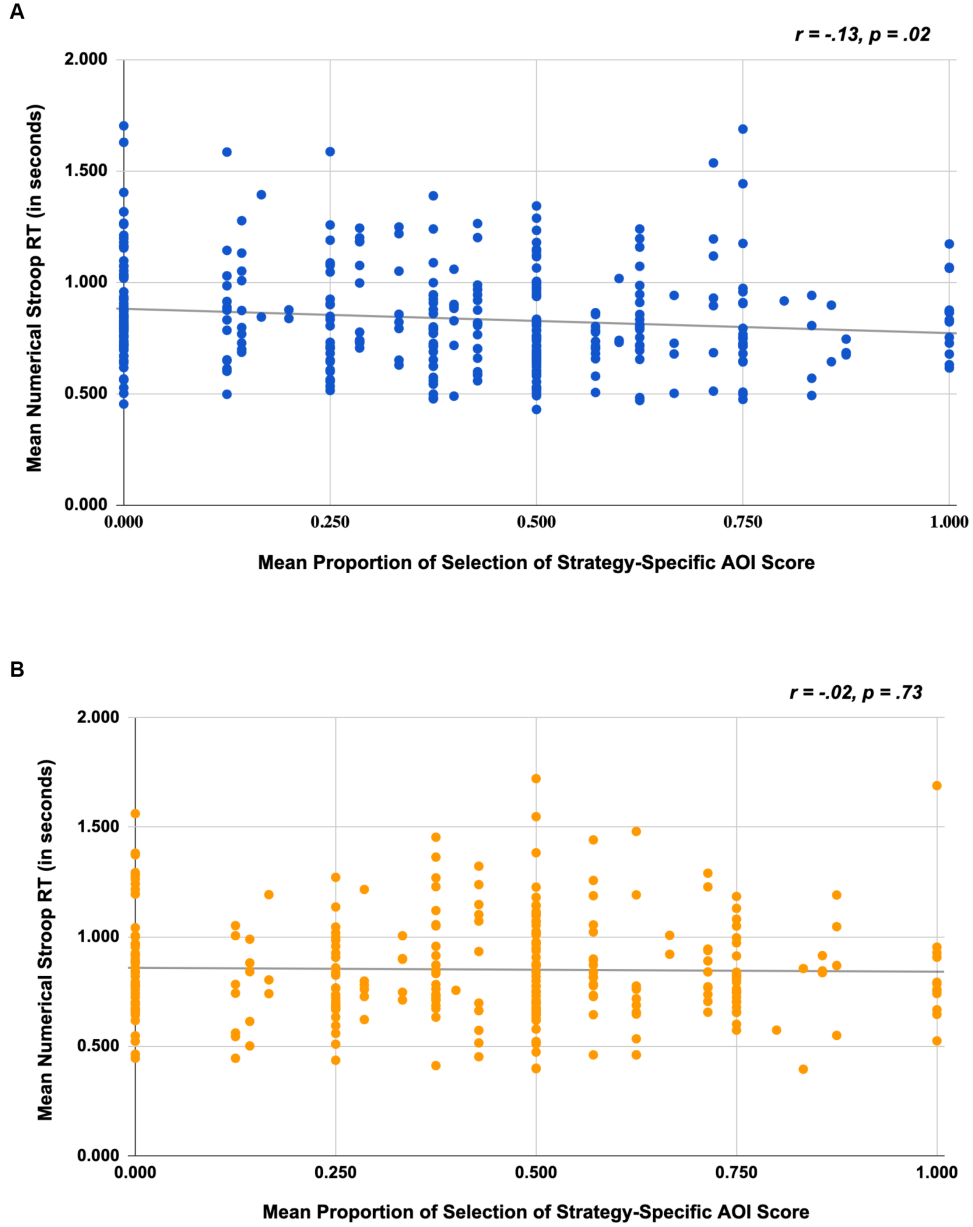
Scatterplots depicting the association between numerical Stroop RT and mean selection of strategy-specific AOI scores for Study 1 **(A)** and Study 2 **(B)**.

To evaluate whether individuals with stronger inhibitory control were also likely to report attending to strategy-relevant AOIs, we conducted a correlation analysis. For Study 1, results revealed a significant negative correlation (*r* = −0.13, *p* = 0.02) suggesting that individuals who have better inhibitory control, marked by shorter RTs, were also more likely to attend to Strategy-Specific AOIs; see Figure 3A. However, in Study 2, the results did not corroborate findings from Study 1 (*r* = −0.02, *p* = 0.73); see Figure 3B.

#### Association between inhibitory control and fraction arithmetic accuracy

**Figure 4:**
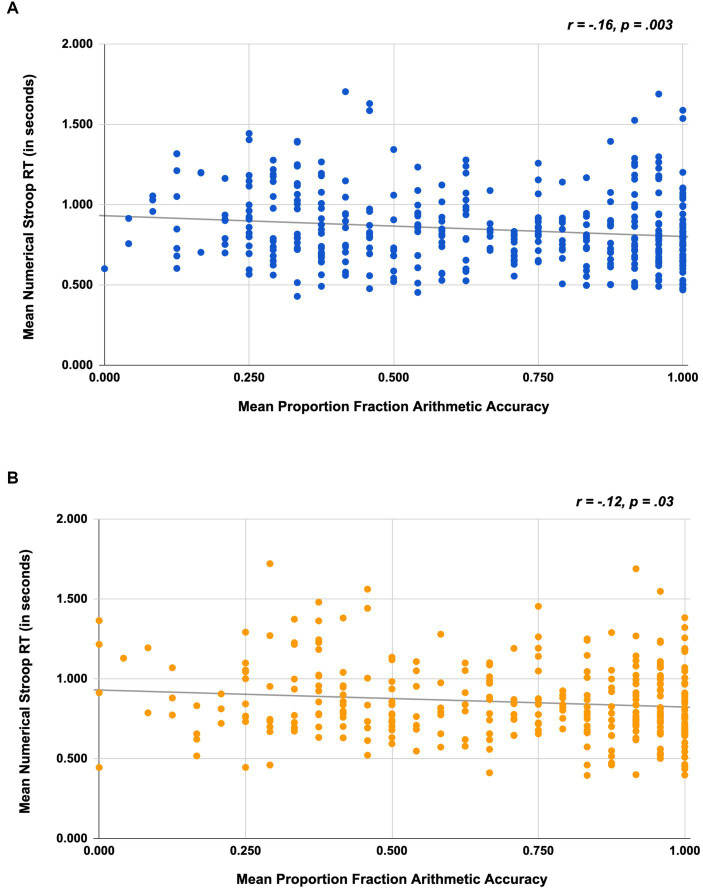
Scatterplots depicting the association between numerical Stroop RT and fraction arithmetic accuracy for Study 1 **(A)** and Study 2 **(B)**.

To assess whether inhibitory control was associated with participants’ math performance, we conducted a correlation analysis in which we investigated the association between participants’ Numerical Stroop RT on the incongruent block (for correct trials only) and their accuracy on the fraction arithmetic problems. In Study 1, results indicated that on average, participants who had better inhibitory control (i.e., shorter RTs) also exhibited greater accuracy on the fraction-arithmetic problems (*r* = −0.16, *p* = 0.003); see Figure 4A. The results were analogous for Study 2. On average, participants who had better inhibitory control (i.e., shorter RTs) also tended to exhibit greater accuracy on the fraction-arithmetic problems (*r* = −0.12, *p* = 0.03); see Figure 4B.

#### Exploratory analyses: orthography and the self-report attention measure

Participants’ cultural context may also impact attention allocation patterns. For example, the orientation of a participant’s orthography may influence where individuals direct their attention. English orthography is oriented left-to right; thus, participants in the present study may be inclined to attend to specific fraction elements such as Numerator 1 (top left) as they would be expected to read and process the equation from left to right (see Opfer et al., 2010 for discussion of spatial-numeric associations). In both Study 1 and Study 2, the orientation of the English orthography may have influenced participant responses to some extent because heat map selection for participants of Numerator 1 (top left AOI) were selected with more regularity than Numerator 2 (top Right); Study 1: *M* = 20%, SD = 29% vs. *M* = 5% SD = 14% and in Study 2: *M* = 20%, SD = 31% vs. *M* = 4% SD = 11%, respectively.

To investigate this hypothesis further, we conducted exploratory analyses using a stricter, more conservative, operation-specific strategy score. This stricter variable was created to measure participants’ tendency to choose only the most vital strategy-related element, given English-speaking participants read from left-to-right and top-to-bottom. Thus, for the Conservative operation-specific strategy, a correct response was defined as selecting Denominator 1 (bottom left) for addition/subtraction problems, Numerator 1 (top left) for multiplication, and Denominator 2 (bottom right) for division. All other AOI selections were scored as incorrect.

##### Association between the conservative operation-specific strategy AOI selection and inhibitory control

Results of the exploratory analyses were largely consistent with the original findings. In Study 1, participants with stronger inhibitory control (shorter RT) were still more likely to attend to the Conservative strategy-relevant AOIs (*r* = −0.21, *p* < 0.001). In Study 2, inhibitory control (i.e., shorter RT) was not significantly correlated with participants’ reports of attending to the Conservative strategy-relevant AOIs (*M* = 34%, SD = 25%; *r* = −0.03, *p* = 0.57).

##### Association between the conservative operation-specific strategy AOI selection and fraction arithmetic accuracy and confidence judgments

Across both Study 1 and Study 2, participants who reported attending to the Conservative strategy-relevant AOIs also tended to have higher fraction arithmetic accuracy for both critical trials (Study 1: *r* = 0.18, *p < 0*.001; Study 2: *r* = 0.28, *p < 0*.001) and for all trials (Study 1: *r* = 0.19, *p* < 0.001; Study 2: *r* = 0.29, *p* < 0.001).

Similarly, in Study 1, participants who selected the Conservative strategy-relevant AOIs tended to report greater confidence in their fraction arithmetic answers for critical trials (*r* = 0.10, *p* = 0.05); however, the correlation was not significant when looking at the association with participants’ confidence judgments across all trials (*r* = 0.096, *p* = 0.07). Results for Study 2 were largely analogous. The correlation between the selection of Conservative strategy-relevant AOIs and confidence judgments of participants’ fraction arithmetic answers was positive and in the expected direction for both critical trials (*r *= 0.11, *p* = 0.06) and across all trials (*r* = 0.11, *p* = 0.06); however, neither correlation was statistically significant.

## Discussion

The present work explored whether self-reported attention (via heat maps) to fraction arithmetic components is related to inhibitory control, fraction arithmetic accuracy, and participants’ confidence judgments. Additionally, we investigated whether inhibitory control is related to fraction arithmetic performance. We also investigated the consistency of the findings through a replication study (Study 2). Several consistent findings emerged (see Table 3). Across both Studies 1 and 2, and in line with our hypotheses and prior work (Siegler et al., 2011; Braithwaite et al., 2017, 2019; Sidney and Alibali, 2017; Braithwaite and Siegler, 2018b; Sidney et al., 2019a), fraction arithmetic accuracy varied as a function of operation type with participants exhibiting the lowest accuracy rates for division problems and the highest accuracy rates for subtraction problems. Further, participants who were more accurate on the fraction arithmetic problems also tended to be more confident in their answers and demonstrated stronger inhibitory control (as indexed by shorter RTs on the Numeric Stroop Task). This pattern of results aligns with our hypotheses and the prior literature on fraction arithmetic and EF (St Clair-Thompson and Gathercole, 2006; Siegler and Pyke, 2013).

**Table 3:** Displays key findings across Study 1 and Study 2.

		Study 1	Study 2
Fraction arithmetic accuracy	Effect of operation type	Sig.	Sig.
	Pairwise comparisons: division vs. all other operation types	Sig. lower accuracy	Sig. lower accuracy
	Pairwise comparisons: subtraction vs. all other operations	Sig. higher accuracy	Sig. higher accuracy
	Pairwise comparisons: addition vs. multiplication	Sig. higher accuracy	NS
Self-report attention measure (AOIs) and fraction arithmetic accuracy	Correlation: Selection of Operation AOI and Fraction Arithmetic Accuracy – *Critical Trials*	NS correlation	NS correlation
	Correlation: Selection of Operation AOI and Fraction Arithmetic Accuracy – *All Trials*	NS correlation	NS correlation
	Correlation: Selection of Strategy relevant AOI and Fraction Arithmetic Accuracy – *Critical Trials*	Sig. + correlation	Sig.+ correlation
	Correlation: Selection of Strategy relevant AOI and Fraction Arithmetic Accuracy – *All trials*	Sig. + correlation	Sig. + correlation
Confidence judgments (CJ)	Correlation: CJ and Fraction Arithmetic Accuracy	Sig. + correlation	Sig. + correlation
	Correlation: CJ and Fraction Arithmetic Accuracy – within each operation type	Sig. + correlation	Sig. + correlations
	Correlation: CJ and Self- Report Attention Measure (strategy-relevant AOI) – *Critical trials*	Sig. + correlation	NS correlation
	Correlation: CJ and Self- Report Attention Measure (strategy-relevant AOI) – *All trials*	Sig. + correlation	NS correlation
Inhibitory control (numerical Stroop RT)	Correlation: Stroop RT and Fraction Arithmetic Accuracy	Sig. − correlation	Sig. − correlation
	Correlation: Stroop RT and Self- Report Attention Measure (strategy relevant AOI)	Sig. − correlation	NS

Highlighted cells indicate consistent findings across Study 1 and Study 2. Darker shading indicates consistent significant results across Study 1 and 2. Lighter shading indicates non-significant findings that are consistent across Study 1 and 2.

This work also provides initial support for heat maps as a beneficial tool that can be used to conveniently and efficiently measure adults’ self-reported attention to fraction arithmetic components during remote data collection. It will be important in future research to assess whether this measure can also be used reliably with children. Here we measured participants’ self-reported attention to the Operation AOI and Strategy-Specific AOIs via mouse click as an index of their problem-solving strategies. Contrary to our hypothesis, across both studies, participants did not consistently select Operation AOIs across trials (i.e., sometimes participants selected the Operation AOI first and sometimes they did not), and the frequency with which participants selected the Operation AOIs was unrelated to their fraction arithmetic scores. This finding was unexpected, as we anticipated that participants would first attend to the Operation AOIs to determine which problem-solving strategy they should deploy. Our *post-hoc* hypothesis is that the Operation AOIs may not have been a salient cue as participants were informed that they would be answering six problems per operation type, and the presentation order of the fractions was blocked by operation. This design choice was made because in the larger parent study, participants were told they would need to solve 24 fraction arithmetic problems (six problems per operation), and they made predictive judgments about the number of problems they would solve correctly. In future work, researchers could modify the presentation order of the stimuli, for example by interleaving problems, to assess whether participants are more likely to attend first to Operation AOIs when they do not have foreknowledge of the operation type.

Critically, participants’ mean selection of Strategy-relevant AOIs was positively correlated with their fraction arithmetic accuracy across both Studies 1 and 2. In other words, participants who selected fraction components that were aligned with the operation type (addition, subtraction, multiplication, division) also tended to be more accurate on fraction arithmetic problems. Due to the limited number of items administered per operation type, we are unable to ascertain whether these findings also hold *within* each operation type (i.e., addition, subtraction, multiplication, division). This will be an important question to address in future research given differences by operation type in participants’ fraction arithmetic accuracy observed in the present study as well as in prior research (Siegler et al., 2011). Nevertheless, these findings provide preliminary support for the use of heat maps as one tool that can be used to capture adults’ self-reported attention to fraction arithmetic components.

This work points to the exciting possibility that heat maps may be a useful tool researchers can deploy to better understand the relationship between attention, strategy selection, and math performance. Heat maps have the advantage of being readily deployed online and can conceivably be used with a wide age range of participants. In comparison to verbal strategy reports, the linguistic response demands of heat maps are attenuated as participants’ response is simplified to a mouse click. There are also logistical advantages to heat maps such as minimal time required to score participant responses compared to verbal strategy reports that have heavy demands on researchers’ time for scoring and the time needed to establish strong inter-rater reliability.

Eye tracking also has a number of advantages including the potential to elucidate the processes underlying mathematical thinking (Strohmaier et al., 2020). Since eye-tracking is an implicit measure it reduces metacognitive and linguistic response demands. Thus, it is perhaps not surprising that eye tracking has become an increasingly popular research methodology in math cognition (for review see Strohmaier et al., 2020). However, eye-tracking also has limitations. As noted previously, eye-tracking can be difficult to deploy online and broader participation may be limited given the heightened participant demands (e.g., uploading their own data, access to webcam, reliable internet connection). Data loss is also a significant and costly issue that often necessitates oversampling (Bott et al., 2017; Semmelmann and Weigelt, 2018 Strohmaier et al., 2020). However, given that every measure, including heat maps, has both strengths and limitations it may be fruitful to use heat maps, not in lieu of strategy reports and eye-tracking, but as a converging measure that can be leveraged to triangulate participants’ attention and strategy selection.

This study also indicated that inhibitory control, a component of EF, is related to participants’ fraction arithmetic performance, such that individuals who exhibited stronger inhibitory control tended to obtain higher fraction arithmetic accuracy. This finding is aligned with our hypothesis and also contributes to the prior literature by employing a domain-specific measure of inhibitory control (Numerical Stroop). Greater inhibitory control may be particularly beneficial in the context of fraction arithmetic as this cognitive skill may enable individuals to “overcome” the whole-number bias by helping them inhibit whole number strategies that are largely automatized due to the extensive experience individuals have with whole numbers (Siegler et al., 2011; Fitzsimmons et al., 2020b; Leib et al., 2023). Indeed, when dealing with fractions, generalizing some whole-number strategies can be counterproductive. For example, when fractions have the same numerator, bigger denominators reflect smaller magnitudes (e.g., ½ vs. 1/16), which counters individuals’ extensive experience with whole numbers (e.g., 2 < 16).

Further, in Study 1, greater inhibitory control was also related to participants’ attention to strategy-relevant fraction arithmetic components, and thus may point to a potential mechanism by which EF influences math performance. It is possible that greater inhibitory control could enable an individual to more readily and successfully inhibit prepotent whole number strategies, thus providing the necessary cognitive capacity to attend to strategy relevant cues within the fraction problems. It is also important to note that the significant association between inhibitory control and attention to strategy-relevant AOIs was not corroborated in Study 2. It is unknown whether the significant correlation in Study 1 is a Type 1 error or whether the correlation in Study 2 is a Type II error. Thus, future research is needed to adjudicate between these possibilities.

It is also interesting to note that participants’ attention to fraction components was highly variable across trials with less than a quarter of participants consistently reporting that they attended to relevant fraction components or the operation. Future research should include additional trials per operation type to ascertain whether this lack of stability is also evident within each operation type. Additionally, by including more test items per operation, it will be possible to test the hypothesis that different operation types will exert variable demands on participants’ inhibitory control. For example, addition and subtraction problems might require greater inhibitory control to inhibit competing strategies for common denominator problems as compared to problems with non-common denominators. Thus, investigating differences in AOI strategy selection both across and within operation types (i.e., common vs. uncommon denominators) and in turn, differences in the association strength between inhibitory control and AOI strategy selection will be an important direction for future work.

As noted previously, prior work has found that individuals’ confidence judgments generally align with their performance (Siegler and Pyke, 2013). It is reasonable to predict that confidence judgments might also be related to patterns of attention allocation as individuals who have more knowledge of the procedures or heuristics necessary for successfully solving fraction arithmetic problems and thus attend to the strategy-relevant fraction arithmetic components may be more confident in the accuracy of their solution. Similarly, individuals may know that fractions necessitate specific procedures, but realize they do not know the procedures (or have forgotten them), resulting in lower levels of confidence and less attention to strategy-relevant fraction components. In line with our reasoning, in Study 1, participants’ confidence judgments were positively correlated with their self-reported attention to strategy relevant AOIs. However, in Study 2 the correlations, although positive and similar in magnitude, were not statistically significant. Factors underlying these divergent results should be explored in future research in order to elucidate whether people’s confidence is underpinned in part by knowledge of relevant fraction strategies.

Despite the initial promise of this work, there are several limitations that should be noted and addressed in future work. First, it will be important to assess the validity of this self-report measure of attention (i.e., mouse clicks on heat maps) given the level of metacognitive sophistication the task might require of participants to accurately report where they attended first. To the best of our knowledge, in the prior literature, it is unknown what components of a problem individuals attend to first, or the order in which they attended to them. In the strategy-report literature, both children and adults can provide verbal explanations of how they solved fraction arithmetic problems (Siegler et al., 2011; Siegler and Pyke, 2013). However, their verbal descriptions do not necessarily include information as to which aspects of the problems they attended to first, or the sequence in which they attended to each part of the problem. Eliciting such information may require explicit prompting. Additionally, it is important to acknowledge that the level of detail individuals provide in their strategy reports is variable and may reflect an individual difference. In our future research, we plan to triangulate heat maps and verbal reports as well as leverage eye-tracking technology to assess the potential alignment of participants’ self-reported attention to fraction components with more objective and implicit measures (see Wall et al., 2015 for related preliminary results). Future research should also investigate the extent to which characteristics of the stimuli may influence participants’ attention allocation patterns. For example, does the operation symbol, which was presented in the middle of the screen, capture participants’ attention, similar to a fixation point used in various cognitive tasks?

Third, participants were asked to report via mouse click the first place they attended. In future research, we aim to capture participants’ problem-solving steps sequentially to identify critical points for intervention. That is, interventions may be most successful when they are applied at the points in the problem-solving process in which strategies are erroneously applied.

Lastly, the Numerical Stroop Task employed in the present study (Fitzsimmons et al., 2020b) is a domain-specific measure of inhibitory control. In some prior research, a stronger correlation was found between mathematics achievement and inhibitory control when using a domain specific inhibitory control measure - for instance, inhibitory control tasks that included numerical information compared to tasks that incorporated non-numerical information (see Gilmore et al., 2015). Incorporating both domain-general and domain-specific inhibitory control measures in future research will allow scientists to more fully explore potential domain-specific effects in fraction arithmetic. Additionally, the task impurity problem is ubiquitous and the Numerical Stroop task deployed in the present study inevitably taps into other cognitive skills such as working memory and processing speed, in addition to inhibitory control. It is important in future research to include a comprehensive individual difference assessment battery in which multiple measures of each construct could be used to help address the task impurity problem (Miyake et al., 2000b) and to elucidate the unique contributions of different components of executive function (i.e., working memory, shifting, inhibitory control) as well as processing speed in order to more fully explore the importance of inhibitory control and other related cognitive skills for fraction arithmetic accuracy and attention to strategy-relevant fraction arithmetic components.

## Conclusion

This work provides a foundation to more fully explore how heat maps can be leveraged as a beneficial tool to conveniently and efficiently measure self-reported attention to fraction arithmetic components, an approach that can be used both in-person and importantly during remote data collection to capture participants’ problem-solving steps. Such an approach could be leveraged to detect error patterns and in turn inform interventions. Indeed, this line of work has potential to inform educational interventions and instructional approaches that may help improve children and adults’ understanding of fractions and ultimately improve achievement in mathematics. The benefits of such an intervention have the potential to produce a positive cascade given associations between mathematical competency and life success (National Mathematics Advisory Panel, 2008; Bjälkebring and Peters, 2021).

## Data availability statement

The raw data supporting the conclusions of this article will be made available by the authors, without undue reservation.

## Ethics statement

The studies involving humans were approved by Kent State University Institutional Review Board (IRB). The studies were conducted in accordance with the local legislation and institutional requirements. The participants provided their written informed consent to participate in this study.

## Author contributions

KG, CT, CF, and JT: conceptualization. KG, CT, FK, YI, CF, and JT: data curation. KG, FK, and YI: analysis. KG, CT, FK, YI, CF, and JT: writing—original draft. KG, CT, FK, YI, CF, and JT: writing—reviewing and editing. All authors contributed to the article and approved the submitted version.
