# Supplementary material for: Attending to what’s important: what heat maps may reveal about attention, inhibitory control, and fraction arithmetic performance
Source: Front Psychol. 2023 Nov 1;14:1210266. doi: 10.3389/fpsyg.2023.1210266 (PMC10646336; doi:10.3389/fpsyg.2023.1210266)
Supplement: Supplementary file 1 [file Data_Sheet_1.docx]

**Supplementary Materials**

**Detailed Description of the Scoring Procedure for the Fraction Arithmetic Problems**

The following description details the scoring procedure utilized to assess the accuracy of participants’ responses on the fraction arithmetic problems in both Study 1 and Study 2. All non-responses were scored as incorrect. Additionally, participants were not asked to reduce their answer to the lowest term and therefore, accurate answers that were not reduced were deemed correct as were accurate answers that were written as decimals. Accurate answers entered in the numerator response box with a denominator of 1 were scored as correct. Similarly, accurate answers entered in the numerator response box with a denominator of N/A or a denominator that was left blank were also scored as correct as blanks and N/A responses were inferred to represent a 1. Additionally, if a participant entered a mixed fraction with appropriate text explanation into either box, it was scored as correct if the value upon solving the mixed fraction matched the correct answer. Responses with meaningless text alongside numbers (e.g., 3x) and/or multiple responses in each response box (e.g., 3 (27)) were scored as incorrect. However, a division sign (/) typed alongside the correct numerator (e.g., 3/) was still scored as a correct response as it was inferred to be the division sign between a numerator and denominator.
