# Supplementary material for: Attending to what’s important: what heat maps may reveal about attention, inhibitory control, and fraction arithmetic performance
Source: Front Psychol. 2023 Nov 1;14:1210266. doi: 10.3389/fpsyg.2023.1210266 (PMC10646336; doi:10.3389/fpsyg.2023.1210266)
Supplement: Supplementary file 2 [file Data_Sheet_2.docx]

**Appendix**

Table A1. Table of arithmetic problems included in both studies.

| Numerator | Denominator | Operation | Numerator | Denominator |
| --- | --- | --- | --- | --- |
| 27 | 45 | + | 9 | 45 |
| 3 | 5 | + | 1 | 5 |
| **3** | **5** | **+** | **4** | **5** |
| **2** | **3** | **+** | **3** | **5** |
| 9 | 36 | + | 27 | 45 |
| 1 | 4 | + | 3 | 5 |
| 3 | 5 | - | 1 | 5 |
| 27 | 45 | - | 9 | 45 |
| **2** | **3** | **-** | **3** | **5** |
| 27 | 45 | - | 9 | 36 |
| **4** | **5** | **-** | **3** | **5** |
| 3 | 5 | - | 1 | 4 |
| 3 | 5 | x | 1 | 5 |
| **3** | **5** | **x** | **4** | **5** |
| 9 | 36 | x | 27 | 45 |
| **2** | **3** | **x** | **3** | **5** |
| 27 | 45 | x | 9 | 45 |
| 1 | 4 | x | 3 | 5 |
| 3 | 5 | $\div$ | 1 | 4 |
| 3 | 5 | $\div$ | 1 | 5 |
| **2** | **3** | $\div$ | **3** | **5** |
| 27 | 45 | $\div$ | 9 | 45 |
| 27 | 45 | $\div$ | 9 | 36 |
| **4** | **5** | $\div$ | **3** | **5** |

Note. Operations in bold and underlined are critical trials that included heat maps and the AOI attention measure.
